# Potential Application of Camel Milk as a Therapeutic Ingredient in Bath Soaps and Shampoos

**DOI:** 10.1155/2024/4846339

**Published:** 2024-08-23

**Authors:** Elly Oginga, Julius Toeri, Eunice Marete, Joshua Arimi

**Affiliations:** ^1^ Department of Physical Sciences Meru University of Science and Technology, Meru, Kenya; ^2^ Centre of Excellence in Camel Research Meru University of Science and Technology, Meru, Kenya; ^3^ Department of Food Science Meru University of Science and Technology, Meru, Kenya

## Abstract

The increasing worldwide market for natural-ingredient-based cosmetic toiletries is fuelled by the awareness of the dangers of synthetic cosmetics and benefits of natural-based cosmetics on the skincare and management of skin disorders. Besides naturally formulated cosmetics being biodegradable, they also contain ingredients which are chemically beneficial to human skin. Milk-based cosmetics are very promising since milk is rich in essential components such as lactoferrins, vitamins, and lactic acids, which have shown therapeutic properties against disorders such as skin cancer, acne scars, and dandruff. One of the milk that is very promising in the cosmetics industry is the camel milk. Currently, there is limited information in literature regarding the use of camel milk in cosmetics and their benefits. Camel milk stands out from bovine milk following its unique therapeutic properties and chemical composition, making it a potential ingredient for skincare and haircare products such as bath soaps and shampoos. The aim of this paper is to review the available literature on camel milk composition and evaluate the contribution of camel milk constituents to cosmetics.

## 1. Introduction

Globally, cosmetic usage has become a routine in human life due to its benefits to skincare, haircare, and makeup [1]. For instance, the COSMILE Europe report indicates that about 450 million of the European population are utilizing personal grooming products such as shampoo, bath soap, shower gel, hair conditioner, and skincare creams daily. Also, the cosmetics industry has been growing yearly with an annual growth rate of 7%, and the cosmetics global market is projected to reach US $800 billion by 2024 [2].

According to several studies, cosmetics are vital in modifying the human body outlook, enhancing the body's pleasant smell, and protecting the body against bacterial attack [3]. Antioxidant-based cosmetics are found to have a range of benefits to humans such as making skin to look younger and anti-inflammatory [4]. The authors in [5] added that “natural cosmetics” such as herbal face cream, soaps, and shampoos contain essential nutrients, vitamins, and minerals that protect humans against some diseases such as dandruff and acne.

The authors in [6] reported that an increased demand for natural cosmetics is due to the increased awareness of the effects of the chemicals present in the synthetic cosmetics. For instance, 1,4-dioxane, a common ingredient in shampoos, is reported to cause breast and skin cancer [7]. Synthetic cosmetic formulations contain colorants, which are aromatic ring and azo compounds that consequently causes side effects such as allergies and asthma to consumers [8]. In addition, in many countries, toxic cosmetic ingredients such as hydroquinone, paraben, and toluene are banned; clinical studies showed that paraben increases the chances of skin cancer, toluene results in allergies, while hydroquinone causes ochronosis [9–11]. Moreover, synthetic cosmetics are made up of heavy metals such as lead and cadmium to enhance color due to their ability of splitting their d-orbitals that allow electrons to transit from low to high energy orbitals [10]. However, these heavy metals expose consumers to disorders such as cancer [12]. Following the abovementioned side effects of synthetic cosmetics and increased awareness of the pollution effects of petrochemicals, users have shifted to organic or herbal cosmetics due to their minimal side effects [13, 14]. Avocado oil, neem, and aloe vera have been profoundly used in formulations of soaps and shampoos owing to their antifungal properties as well as their capability to battle oxidative stress [14].

Recently, milk has become a good ingredient for skincare cosmetics made with natural products as it is loaded with casein and lactoferrin proteins which have antifungal and anticancer properties [15]. In addition, dairy milk is a better component used in skincare cosmetics as it is reported to contain lactic acid for which *in vitro* experiments point out that it exfoliates dead cells from the skin and promotes the growth of new cells [16, 17]. Notably, in Indonesia, high-quality cosmeceuticals for skin and haircare are crafted using goat's milk [18]. Lactic acid contained in goat milk moisturizes and soothes the skin in addition to shedding off the dead cells and promoting new cell growth.

Camel milk, the main source of livelihood for Arid and Semiarid lands' (ASALs) communities, especially herders on long grazing periods [19], is reported as a rich source of *α*-hydroxy acids [20], which offers solutions to skin diseases such as dermatitis and acne. This places camel milk as a promising ingredient for cosmetics made from natural products, offering potential benefits against the mentioned skin diseases.

Kenya is ranked globally as the leading producer of camel milk as it records approximately 1.165 MMT of camel milk by volume every year [21]. However, despite Kenya being on the global map as a leading producer of camel milk, it is unfortunate that camel milk is underutilized due to unawareness of its cosmetic use and the market value. Also, little is documented on the application of camel milk in the development of cosmetic products.

The focus of this paper is therefore to review recent literature on the potential application of camel milk in the production of toiletries such as bath soap and shampoo.

## 2. Trends in Cosmetics from Natural Products

The use of natural products in developing cosmetics has attracted interest from consumers considering that natural products have minimal side effects [2].

The authors in [22] reported that apigenin (4,5,7-trihydroxyflavone), a flavonoid synthesised from aromatic herbs and fruits, showed protective effects in reducing skin tumors. In cancer cells, apigenin (4,5,7-trihydroxyflavone) causes apoptosis or programmed cell death. In this process, the reactive oxygen species (ROS) pathway is activated, which lowers the generation of ROS and causes cell shrinkage, protein breakdown, and the inhibition of tumor growth by 2-deoxyribonucleic acid (DNA) [23].

In addition, porphyra-334, an alkaloid isolated from sea cucumbers and red algae, is capable of softening skin by removing skin wrinkles. Wrinkles and photoaging are associated with collagen degradation and UV irradiation, respectively. Porphyra-334 inhibits the production of matrix metalloproteinase (MMP) enzymes that break down collagen and enhance the production of collagen and elastin in skin cells exposed to UV radiation [24].

According to the authors in [25], the market demand for “green cosmetics” such as skincare products has grown tremendously since besides enhancing beauty they also promote environment conservation. Today, the use of cosmeceuticals has emerged in the field of cosmetics in which cosmetics are formulated with bioactive ingredients that provide both therapeutic and cosmetic benefits to skin and hair.

The authors in [26] highlighted examples of natural products and their sources (Table 1) that have made their utilisation in cosmeceutical formulations. In addition, the researchers also noted rapid growth in personal care products as compared to other sector of natural products.

Animal products especially milk have recently found a wide application in cosmeceutical formulations [15]. Milk possesses proteins which are antifungal, antioxidant, anticancer, and antibacterial [27]. Research carried out by the authors in [28] reported that soap formulated using goat milk has potent antimicrobial activity against microorganisms such as *Staphylococcus aureus* and *Escherichia coli*. Similarly, another study carried out by the authors in [29] reported that the addition of goat milk to shampoo improves its physicochemical properties such as conditioning performance, cleaning capacity, and foaming ability.  Table 2 presents a summary of dairy-based cosmetic products.

Camel milk's health and therapeutic properties, as well as its higher concentration of bioactive components than ruminant (goat and cow) milk, make it a promising ingredient for natural cosmetics, as shown in Table 2.

## 3. Chemical Composition of Camel Milk and Their Cosmetic Potential

Numerous studies have indicated that the chemical composition of camel milk relies on variables such as seasonal variations, camels' health conditions, nutrition, management, geographical location, and feeding regime [33–35]. For example, the authors in [36] reported that between the years 1989 and 2009, C*amelus dromedarius* milk was found to contain on average 3.5% fat, 3.1% protein, and 4.4% lactose. A decade later (2019) in Uzbekistan [37] on their comparative analysis of camel composition over winter and summer seasons found that camel milk contents are relatively lower in the summer period, and it was suggested to be attributed to changes in the food supply. It is reported that unlike bovine milk, Camelus dromedarius milk fat has a little content of carotene and minimal concentration of short-chain fatty acids. Also, the authors in [38] reported that camel milk contains essential proteins such as lactoferrin and immunoglobulins which have antibacterial, antiviral, antifungal, and antimicrobial properties; hence, camel milk protects skin against infection. A study carried out by the authors in [40] reported that camel milk is rich in *α*-hydroxy acids compared to bovine and goat milk. Also, the researchers established that camel milk has a high content of magnesium and zinc minerals compared to bovine milk.

Camel milk is rich in liposome, which is a potential ingredient of antiaging cosmetic products [39]. For instance, in Korea, a study explored the use of camel milk in cosmetics by preparing liposomes containing camel milk and testing them on human skin fibroblasts. The results showed that the camel milk liposomes increased collagen and hyaluronan synthase-3 (HAS-3) gene expression in a concentration-dependent manner while inhibiting elastase activity and matrix metalloproteinase-1 (MMP-1) gene expression. In addition, the liposomes were found to regenerate ultraviolet B (UVB) damaged fibroblasts. These findings suggest that camel milk liposomes have the potential as an antiaging cosmetic ingredient [40].

Likewise, camel milk is an affordable cosmeceutical that does not cause any negative systemic or cutaneous reactions because of its constituents, which include water, peptides, alpha-hydroxy acids, ascorbic acids, polyunsaturated fatty acids, and micronutrients [41]. The study also concluded that camel milk is a potential ingredient of skin softener, antiaging, antiwrinkle, and photoprotective products.

### 3.1. Alpha-Hydroxy Acids in Milk

Alpha-hydroxy acids (AHAs) are weak organic acids in which the first carbon atom (*α*-carbon) from the carbonyl carbon is attached to the hydroxyl group [42]. Alpha-hydroxy acid is naturally obtained from foods and milk and it constitutes glycolic acid from sugarcane, citric acid from fruits, and lactic acid from milk [43].

According to the authors in [44], lactic acid (2-hydroxypropanoic acid) is widely used in cosmetic formulations for its antiaging, lightening, and moisturizing properties. For instance, antiaging creams, age-spot removers, toners, and lactic acid powders are some of the commercial products that utilize lactic acid to enhance skincare [45]. Similarly, Baldo et al. [46] investigated the clinical efficacy of a lactic acid-containing cream on patients with mild to moderate acne. The study involved 248 patients who applied the cream twice daily for 60 days. The results showed a high efficacy rate of 92.3% in acne treatment.

The researchers further reported that lactic acid molecule exists in two racemic mixtures of D (−) and L (+), and that out of the racemic mixture, the L (+) enantiomer is more active biologically. In addition, L (+) lactic acid ((S)-hydroxy propanoic acid) is a collagen stimulator [47]. D (−) lactic rotates a plane of polarized light to the right, while L (+) lactic rotates a plane of polarized light to the left.

**Figure 1:**
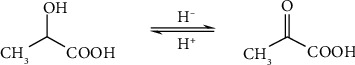
Deprotonation of lactic acid in aqueous medium source [44, 50].

The authors in [48] observed that lactic acid acts as a suppressant of enzyme tyrosinase activities, an enzyme that regulates skin coloration [49]; hence, lactic acid plays a role in brightening of the skin. In the aqueous medium of the epidermis, the hydroxyl group of the lactic acid undergoes a deprotonation reaction forming pyruvic acid (Figure 1) [44]. The pyruvic acid formed stimulates fibroblast to produce collagen and elastin which give skin a youthful outlook and repairs the skin [50, 51].

A study carried out by the authors in [52] to compare the therapeutic activities of a 2% concentration of both salicylic and lactic acids in the treatment of 46 people having acne vulgaris found that lactic acid is more effective than salicylic acid. Similarly, another clinical study performed by the authors in [53] confirmed that 88% lactic acid gel/face wash is particularly good in the treatment of acne scars. According to the authors in [39], the lactic acid present in camel milk enhances the exfoliation of dead skin cells through the breakdown of sugars that hold skin cells together, and consequently, cell growth is facilitated.

### 3.2. Casein Protein in Milk

Casein is a milk protein responsible for the white color and is the main protein present in camel milk and makes about 87% of the protein [54]. Casein is made up of four protein fractions: *α*s1-casein, *α*s2-casein, *β*-casein, and *κ*-casein, and among the casein proteins, *β*-casein is more abundant in camel milk as compared to cow's milk [55]. Also, hydrolysis of *β*-casein is easier compared to *α*s-casein. Casein reacts with sodium ions to form sodium caseinate which is used up to 96.9% in bath oils [56]. Commercial casein skincare products for antimicrobial infections include bath oils and goat milk soap [28]. Also, casein nanofibers containing silver nanoparticles have antibacterial and biocompatible properties, which suggest that they could be used in skincare products [57].

### 3.3. Lactoferrin in Milk

Lactoferrin as an iron-binding glycoprotein protects the human body against microbial and inflammatory infections [58]. Bovine lactoferrin structure contained about 696 amino acids [59]. Upon completion of 8 weeks of clinical trials for acne treatment using bovine lactoferrin, the authors in [60] noticed a significant improvement among patients as 30 out of 39 patients involved in the clinical study showed a decrease in acne papules and pustules. Also, a comparative study of the antimicrobial spectrum of both bovine and human lactoferrin revealed strong microbial inhibitory effects of bovine lactoferrin [61]. A range of scientific inquiries has consistently shown that bovine lactoferrin being anticarcinogenic stimulates immune system cells to release cytotoxic agents to hinder tumor cell growth [62–64]. Likewise, an *in vitro* study carried out by the authors in [65] revealed that camel milk lactoferrin has a significant ability to reduce the growth of cancer cells. Camel milk lactoferrin reduces cancer growth by 56% as reported by the authors in [65]. Researchers also added that the anticancer properties of camel milk lactoferrin could be because of its direct cell cytotoxicity and antiangiogenic action which involves cutting off the blood supply to tumor cells.

## 4. Camel Milk as Cosmeceutical Ingredient

### 4.1. Skin Cancer

According to the World Health Organization report [66], cancer prevalence is approximately 18.1 million, with melanoma the skin cancer ranked number 17 out of 33 cancers. Furthermore, based on the increased cancer cases yearly, the report projected that the global cancer cases could double to 27–37 million cancer cases by 2040. Exposure to chemical ingredients in synthetic cosmetics is found to be one of the ways that causes skin cancer since the skin absorbs about 60% of the chemical ingredients [67]. For instance, the authors in [68] reported that most of the shampoos are formulated using nitrosamines which are carcinogenic. Following the escalating global cancer prevalence and the anticipated increase in cancer cases, there is a growing need for ground-breaking approaches in cancer treatment and prevention, including in the field of cosmetics.

Camel milk is promising in the treatment of tumor and skin cancer therapy as it is packed with lactoferrin that *in vivo* and *in vitro* experiments have demonstrated that it is anticancerous [35]. In addition, the authors in [69] also claim that camel milk provides an avenue for inhibition of the growth of breast cancer cells BT-474 cells. Similarly, a study on the anticancer effects of ruminant milk carried out by the authors in [70] found that camel milk has the greatest cytotoxic activities to counter MCF7 breast cancer-causing cells compared to other ruminants' milk. Furthermore, the milk fat globule membrane of the dromedarius camel milk has the ability to eliminate cancer cells [71]. However, it is worth noting that camel milk's potential anticancer benefits are still in the early stages of research. There are few large-scale clinical trials in humans to confirm these effects. In addition, more research is needed to determine the specific mechanisms by which camel milk components interact with cancer cells.

### 4.2. Dandruff and Seborrheic Dermatitis

The global prevalence of *seborrheic dermatitis* stands at 5%, while that of dandruff is about 50% [72, 73]. Dandruff and *seborrheic dermatitis* are allied to itchy and peeling skin. Both dandruff and seborrheic dermatitis are fungal skin diseases that affect the scalp [74]. In addition, skin regions with thick sebaceous glands such as the scalp and face are susceptible to *seborrheic dermatitis* infection [75]. In Elewski and Ravikumar's reports [72, 76], dandruff is caused by the gathering of corneocytes in the scalp and people aged within the range of 15–50 years are susceptible to dandruff as it is the period at which sebaceous glands are active.

*Seborrheic dermatitis* is currently cured using anti-inflammatory steroids; however, the prolonged exposure of skin to steroids is reported to be associated with adverse effects such as peeling of skin and acne [77]. Also, the frequent use of shampoo containing antifungal agents such as salicylic acid is remarkably effective in controlling seborrheic dermatitis [75].

Camel milk can be a promising remedy for both dandruff and seborrheic dermatitis as it is reported to contain casein peptides [78] that are made up of *α*s1, *α*s2, *β,* and *κ* casein which are reported to have antibacterial, antiviral, antifungal, and antioxidant activities [79]. Moreover, Rasheed [80] reported that N-acetyl-*β*-d-glucosaminidase present in camel milk has shown antimicrobial activity. Dromedary camel milk is loaded with natural phospholipids that initiate the healing of skin through the production of collagen.

### 4.3. Acne Scars

According to the authors in [81], more than 90% of the teenage population is susceptible to acne infection and about 13% of the cases persist up to adulthood. The study added that the two types of scars, hypertrophic and keloidal scars, are caused by the accumulation of collagen [82]. The authors reported that alpha-hydroxy acids such as glycolic acid, pyruvic acid, and lactic acid are one of the biochemical peeling agents used in the control and management of acne scars as they aid in the elimination of dead skin. In India, the pilot study on peeling effects of lactic acid on acne scars performed by the authors in [83] found that 92% pure 2-hydroxypropanoic acid (lactic acid) showed an excellent improvement in peeling superficial acne scars and lightening of acne scars among patients with dark skin. In addition, the authors in [84] found 88% pure lactic acid to have greater efficacy in the treatment of acne vulgaris and acne scars in patients with dark skin. *Camelus dromedarius* milk contains *α*-hydroxy acid that smoothens skin through the elimination of dead skin cells, thus being effective for acne treatment [39]. Also, a study carried out by the authors in [85] on the antimicrobial and anti-inflammatory activity of camel milk against *Propionibacterium acne* found that camel milk-derived immune proteins and peptides have antimicrobial and anti-inflammatory activity against the bacteria. The study findings paved the way for the potential use of camel milk in cosmetics to manage acne vulgaris and other skin infections such as seborrheic dermatitis and dandruff.

### 4.4. Tinea Pedis (Fungal Infection on the Foot)

Tinea pedis is a contagious fungal infection of the feet that majorly occurs between the toes and soles [86]. Tinea pedis is caused by too much accumulation of moisture between the toes and it is linked to itching and burning.

Globally, 15% of people have tinea pedis [87]. The authors in [88] carried out a clinical study on patients with tinea pedis and reported that bovine milk lactoferrin has a healing effect. Also, in another clinical trial where 37 selected adults with tinea pedis were given doses of bovine lactoferrin daily for 56 days [89], decreased symptoms were noticed among the patients. One millilitre of camel milk contains about 0.22 mg of lactoferrin which is much higher compared to cow milk [39]. Therefore, camel milk can be a good replacer of bovine milk in the treatment of tinea pedis.

## 5. Use of Camel Milk in Cosmetics

### 5.1. Camel Milk in Body Wash (Bath Soaps)

Camel milk, besides being a source of food, is replacing other milks in cosmetics due to its therapeutic properties [90]. Camel milk is rich in alpha-hydroxy acids such as lactic acid that help in the elimination of dead cells and spots on the skin [39]. The alpha-hydroxy acids are often used in the production of skincare cosmetics products such as bath soap [91].

The authors in [38] reported that camel milk is rich in essential proteins such as lactoferrin and immunoglobulins, which have antibacterial, antiviral, antifungal, and antimicrobial properties; hence, camel milk protects the skin against infection. It is reported that vitamin C, zinc, and magnesium minerals present in camel milk are good antioxidants and hence aid the skin in relieving oxidative stress [90]. Furthermore, camel milk contains lanolin which is a moisturizer that ensures that the skin is hydrated [20]. The therapeutic nature of camel milk on skin health makes it a better ingredient for natural body wash. According to market data [92], camel milk products were valued at USD 7.24 billion in 2023 and are predicted to reach USD 14.42 billion by 2031. Consumers believe that camel milk has more therapeutic and medical characteristics than cow milk, which drives demand. Similarly, the health benefits of camel milk products have been found to enhance the willingness to purchase the products, particularly among people who suffer from lifestyle disorders [93].

### 5.2. Camel Milk in Hair Products

Shampoo as a haircare product serves a dual cosmetic function of cleansing and enhancing hair beauty [94]. Its primary role relies in maintaining scalp and hair hygiene, a function heavily influenced by its formulation [95]. According to the authors in [96], naturally formulated shampoos are on demand as they contain ingredients which are chemically inert and biocompatible to the users. Research has demonstrated that goat milk has been successfully included in shampoos due to its health and therapeutic benefits [31]. According to the study, goat milk shampoo provided good conditioning performance and had a 30-day shelf life period. A study performed by the authors in [97] found that liquid soap formulas with 15% cow's milk were stable for 28 days due to well-balanced ingredients and compatibility within the formula for preventing degradation over the period.

## 6. Conclusion

The shift towards cosmetics made from natural products is caused by the fact that natural cosmetics are biodegradable and have mild reactions to users' skin compared to synthetic cosmetics. Milk, whether from cows, goats, or camels, has emerged as a valuable ingredient in cosmetics, offering numerous advantages. Camel milk stands out due to its unique chemical composition as it contains essential proteins such as lactoferrin and immunoglobulins, which possess antibacterial, antiviral, antifungal, and antimicrobial properties. Camel milk also boasts an abundance of alpha-hydroxy acids (AHAs), such as lactic acid that are involved in exfoliating dead skin cells, stimulating collagen production, and brightening the skin. Moreover, casein, a predominant protein in camel milk, has demonstrated potential in cancer treatment, and lactoferrin exhibits anticarcinogenic properties, making camel milk a promising candidate for cosmeceuticals. Camel milk's potential role in preventing skin cancer, treating dandruff and seborrheic dermatitis, and addressing acne scars makes it an attractive choice for skincare products.

The benefits of camel milk in cosmetics are compelling, but further research is required to fully exploit its potential. Continued scientific research will assist to validate these preliminary findings and optimize the usage of camel milk in cosmetic compositions. Furthermore, studying the market impact of camel milk-based cosmetic products will provide credible data on customer acceptance and profitability. Camel milk may play an important role in the future of cosmeceuticals if its therapeutic benefits are supported by scientific evidence.

## Figures and Tables

**Table 1:** Natural products made from naturally synthesised compounds.

Ingredient	Action	Source	Product in market
Beta-hydroxy acid	Antibacterial	Salicylic acid	OxyMED shampoo and Skin Medica face cream
Centella	Skin conditioning agent, improves skin texture, and reduces stretchmark	*Centella asiatica*	Himalaya neem face wash
Aloe vera	Softens skin	Aloe vera plant	Aloe vera gel and nature's gel
Rosemary extract	Antioxidant and antimicrobial	*Rosmarinus officinalis*	L'Oreal body cream
Boswellia	Anti-inflammatory and antiaging	*Boswellia serrata*	Aromaboswellia and antiwrinkle cream
Neem oil	Antimicrobial	Azadirachta	Himalaya neem face wash

Source: [26].

**Table 2:** Summary of cosmetic products using dairy ingredients.

Product type	Dairy ingredient	Bioactive components	Benefits	Source
Soaps	Goat milk and goat milk cream and cow milk	Fatty acids and proteins, and lactic acid	Antibacterial, exfoliation of dead cells, and moisturizing	[28–30]
Shampoos	Goat milk	Vitamin C and protein	Conditioning and hair growth	[31]
Body lotions	Goat milk	Lactic acid, vitamin C, and protein	Moisturizing, antiaging, and exfoliation	[32]

## Data Availability

Data sharing is not applicable to this article as no datasets were generated or analysed during the current study.
